# Insulin-like growth factor-1 in myocardial ischemia-reperfusion injury: A review

**DOI:** 10.1097/MD.0000000000037279

**Published:** 2024-03-01

**Authors:** Zhenrong Yan, Ziyang Xing, Tingyun Xue, Jiaye Zhao, Guangmei Li, Liwenjing Xu, Qiyu Sun

**Affiliations:** a Department of Clinical Laboratory, Affiliated Hospital of Chengde Medical University, Hebei, China.

**Keywords:** insulin-like growth factor-1, ischemic heart disease, myocardial ischemia-reperfusion, oxidative stress, preconditioning and post-conditioning

## Abstract

Myocardial ischemia-reperfusion injury (MIRI) is a severe damage inflicted on the ischemic myocardium when blood flow is restored, and it commonly occurs in a wide range of cardiovascular diseases. Presently, no effective clinical treatment exists for MIRI. Accumulating evidence indicates that insulin-like growth factor-1 (IGF-1) plays a role in the intricate chain of cardiovascular events, in addition to its well-recognized growth-promoting and metabolic effects. IGF-1, a member of the insulin family, exhibits a broad spectrum of protective effects against ischemia/reperfusion injury in various tissues, especially the myocardium. In particular, earlier research has demonstrated that IGF-1 reduces cellular oxidative stress, improves mitochondrial function, interacts with noncoding RNAs, and activates cardiac downstream protective genes and protective signaling channels. This review aimed to summarize the role of IGF-1 in MIRI and elucidate its related mechanisms of action. In addition, IGF-1-related interventions for MIRI, such as ischemic preconditioning and post-conditioning, were discussed. The purpose of this review was to provide evidence supporting the activation of IGF-1 in MIRI and advocate its use as a therapeutic target.

## 1. Introduction

In developed countries, ischemic heart disease (IHD) stands as a primary cause of mortality and disability, followed by cerebrovascular disease.^[1]^ Moreover, cardiovascular diseases, including myocardial infarction, heart failure, vascular dementia, hypertension, and its complications, rank as the leading causes of death in China. Heart failure results from various forms of cardiovascular diseases, and in 2013, IHD replaced stroke as the leading cause of death.^[2]^ The increasing morbidity and mortality rates associated with IHD pose a serious threat to human health. Recanalization and reperfusion of obstructed vessels through thrombolytic medication, coronary bypass operation, and percutaneous coronary intervention are recognized as the most efficient therapeutic strategies for the treatment of myocardial ischemia.^[3]^ There is evidence that in myocardial ischemia/reperfusion, both ischemia and reperfusion cause damage to cardiomyocytes.^[4,5]^ Therefore, myocardial reperfusion may exacerbate ischemic cardiomyocyte death in patients with myocardial infarction, leading to myocardial ischemia-reperfusion injury (MIRI).^[5,6]^ The pathophysiology of MIRI involves calcium overload, oxidative stress, excessive reactive oxygen species (ROS) production, mitochondrial damage, inflammatory response, cell death, and signaling pathways associated with cell survival.^[7]^ Hence, it is important to develop treatments that target the molecular mechanisms underlying the development of MIRI for the therapy of IHD and protection against MIRI.

Insulin-like growth factor-1 (IGF-1) is a multifunctional polypeptide growth factor primarily produced by the liver.^[8]^ IGF-1 is involved in the modulation of survival, death, proliferation, polarization, and metabolism of many cell types^[9]^ and plays an essential role in regulating cardiac structure and function. IGF-1 promotes cardiac myocyte growth in vitro, and enhances cardiac myocyte differentiation and survival following ischemic injury.^[10]^ Additionally, IGF-1 augments heart performance in laboratory models of heart failure in vivo.^[11]^ The potential therapeutic value of IGF-1 is underscored by the fact that its administration improves cardiac function in patients with heart failure in humans.^[12]^ Collectively, these observations demonstrate that IGF-1exerts a protective effect on the human myocardium. Moreover, multiple lines of evidence suggest that IGF-1 is protective against I/R injury in various tissues, as well as in the heart. Previous studies have shown that IGF-1 can alleviate I/R-induced acute renal failure,^[13]^ restore neurons in severe hypoxic-ischemic brain injury,^[14]^ reduce structural damage during I/R,^[15]^ prevent apoptosis, and promote cardiomyocyte survival.^[16]^ IGF-1 plays a critical role in protecting the heart against MIRI. The stable expression of IGF-1 allows cells or tissues to resist damage in the course of MIRI, protects cardiomyocytes from ischemia-induced injury, and improves patients prognosis. This review aims to provide comprehensive evidence and discussion of the mechanism of action of IGF-1 in MIRI, supporting its adoption as a therapeutic target.

## 2. Biological properties and activities of IGF-1

IGF-1 refers to a type of polypeptides in the insulin family identified in 1957.^[17]^ In 1987, 2 peptides were successfully isolated from plasma and designated as insulin-like growth factors (IGFs) due to their functional and structural similarities to insulin.^[18]^ To date, only 2 members have been identified: IGF-1 and IGF-2. IGFs are growth-promoting peptides that are structurally related to insulin.^[19]^ The biological effects of IGFs necessitate the activation of specific cell surface receptors. For example, the IGF-1 receptor (IGF-1R), a membrane-spanning tyrosine kinase with similar affinity for IGF-1 and IGF-2, is structurally related to the insulin receptor.IGF-1 and IGF-2 interact primarily with IGF-1R. IGF-1 is highly homologous to insulin.^[20]^ The locus encoding IGF-1 is located on chromosome 12. IGF-1 is a key growth-promoting peptide that acts as both an endocrine hormone and autocrine/paracrine growth factor. The IGF-binding protein family consists of 6 different types of proteins, and most IGF molecules bind to one of its family members in the blood and local tissues. These proteins play an instrumental role in regulating global and local IGF signaling because they bind to IGF with an affinity equal to or greater than that of the IGF-1 receptor.^[21]^

## 3. IGF-1 and cardiovascular system

IGF-1 is primarily synthesized and produced mainly under the action of hypothalamic growth hormone and exhibits different biological activities. It demonstrates multiple effects in numerous organs and is associated with the occurrence of a wide range of diseases. The cardiovascular system is a critical target of IGF-1. IGF-1 can mediate multiple processes in cardiomyocytes, including growth, metabolism, autophagy, and apoptosis.^[22,23]^ Studies have indicated that IGF-1 antagonizes apoptosis in rat cardiomyocytes after ischemia-reperfusion injury, in mouse myocardial infarction, and in cultivated rat cardiomyocytes. The effects of IGF-1 against apoptosis were observed at physiologic concentrations, whereas other growth factors do not seem to be inhibit apoptosis in cardiomyocytes, even at pharmacological concentrations.^[10]^ There is evidence that IGF-1R is abundantly expressed in vascular endothelial cells, smooth muscle cells, and cardiomyocytes, which are more sensitive to IGF-1 than to insulin.^[24]^ IGF-1 affects vascular function and atherosclerosis through multiple pathways, including anti-inflammatory, antiapoptotic,^[25]^ and angiogenic effects.^[26]^ Secondly, it stimulates DNA and RNA synthesis, promotes mitosis and mediates cell proliferation and differentiation. Multiple studies have indicate that IGF-1 plays an instrumental role in cardiomyocyte generation. Studies on adult rat myoblasts in culture have suggested that IGF-1 activates DNA synthesis.^[27]^ It was shown that cardiac stem cell division in IGF-1 transgenic mice was induced by the IGF-1 receptor, along with enhanced telomerase activity and retention of a functional cardiac stem cell reserve.^[28]^ Finally, IGF-1 exerts cardioprotective effects through anti-MIRI and antiaging pathways. The phosphatidylinositol 3-kinase (PI3K)/Akt signaling pathway is implicated in the modulation of cell survival and protects multiple organs from I/R damage. Previous research has revealed that IGF-1 may confer cardioprotection and prevent MIRI in vivo by activating the PI3K/Akt pathway in rats.^[29]^ Therefore, IGF-1 is likely a target of MIRI, and it is necessary to explore the regulatory mechanisms and roles of IGF-1 in MIRI.

## 4. Roles of IGF-1 in MIRI

### 4.1. IGF-1 and the inflammatory response

During MIRI, aseptic inflammation develops and activates an immune response, leading to the production of numerous inflammatory cells. After reperfusion of ischemic myocardium, neutrophils, lymphocytes, and macrophages accumulate and migrate to the ischemic region in response to cell adhesion molecules and chemokines, generating an inflammatory response that exacerbates the injury-induced impairments.^[30]^ Oxidative stress and inflammatory responses are strongly associated. In cells activated by inflammation, enzymes that produce oxidants, such as NADPH oxidase and myeloperoxidase are up-regulated, causing oxidative insults to surrounding tissues and triggering further inflammatory responses. Several studies suggest that IGF-1 acts as an anti-inflammatory agent. For example, it has been reported that there is an inverse relationship between serum IGF-1 and IL-6 levels.^[31]^ Oxidative stress is a primary cause of MIRI that induces various downstream destructive factors. When generated in amounts that exceed the body’s ability to remove peroxides, ROS disrupts the balance between the oxidant and antioxidant systems, leading to oxidative tissue damage. MIRI produces excess oxygen free radicals through the mitochondrial respiratory chain, unusual functions of activated neutrophils, and the xanthine oxidase system. Oxidative stress induced by massive ROS is a vital causative factor in the decline of myocardial systolic and diastolic function and cardiomyocyte death.^[32]^ When blood flow is restored, reperfusion causes further tissue damage through the formation of ROS and a resulting imbalance between cell-forming free radicals and the cell’s intrinsic protective mechanisms.^[33]^ Excessive ROS accumulation in cells leads to MIRI. Thus, eliminating excess ROS from cells and attenuating oxidative stress in cardiomyocytes promotes cardiomyocyte survival and reduces the severity of MIRI. In a recent study, both anti-inflammatory and pro-repair are associated with oxidative stress changes in the vasculature, and IGF-1 is anti-atherosclerotic through both mechanisms.^[34]^ Moderate oxidative stress stimulated IGF-1 and IGF-1R expansion in muscle cells. Elevated ROS levels suppress insulin/IGF-1 signaling and induce apoptosis.^[35,36]^ Results from Li et al^[37]^ showed that although IGF-1 deficiency itself seems to disrupt intracellular Ca^2+^ homeostasis and inhibit cardiac contractile function, it is effective in enhancing resistance to oxidative stress-induced cardiac dysfunction and tolerance to stress. Changes in Ca^2+^-regulating proteins and ROS production may have contributed to the overall survival rate. Decreased levels of IGF-1 are accompanied by increased oxidative stress, which is common in advanced age.^[38]^ Hong et al^[39]^ proposed that IGF-1 transmits a survival message that prevents oxidative stress-induced apoptosis in cardiomyocytes through PI3K and extracellular signal-regulated kinase (ERK)-dependent channels and that the protective effect of IGF-1 is related to the suppression of Bax expansion. Circulating IGF-1 has multifaceted antioxidant, anti-inflammatory, and antiapoptotic effects that alleviate the burden of cardiovascular disease in experimental models. Thus, the inhibition of the inflammatory response and oxidative stress associated with MIRI may contribute to the restoration of cardiac function and structure.

### 4.2. IGF-1 and mitochondrial function

Mitochondria have been identified as pivotal regulators of myocardial injury during ischemia and reperfusion. Mitochondria make up more than one-third of the volume of cardiomyocytes and are specialized to provide energetic support for ion homeostasis and excitation–contraction coupling required for the cardiac cycle. Mitochondria are the primary location for aerobic respiration and the primary target of I/R. Thus, mitochondrial dysfunction plays a pivotal role in MIRI.^[40]^ The pivotal factor contributing to tissue damage and subsequently cell death is hypoxia/reoxygenation (H/R), which occurs in somatic cells. Decreased oxygen content during ischemia affects mitochondrial ATP production and increases intracellular Ca^2+^ levels. In cases of critical myocardial ischemia, damage to the electron transport chain primarily occurs during the ischemic period.^[41]^ Reperfusion of mitochondria in the myocardium can lead to mitochondria-driven early injury if they have been damaged by previous severe ischemia. This results in excessive ROS and dysregulation of calcium regulation, causing abnormal infiltration, swelling, and destruction of the mitochondrial permeability transition pore (PTP).^[42]^ PTP opening leads to Ca^2+^ release, elevated ROS levels, cessation of ATP synthesis, mitochondrial depolarization, and oxidation of proteins and lipids within it, leading to matrix swelling in vitro. In turn, matrix swelling causes cytochrome c mobilization, rupture of the outer mitochondrial membrane, and eventually the release of pro-apoptotic proteins such as endonuclease G and cytochrome c.^[43,44]^ The opening of the PTP further compromises cellular energetics and increases ROS levels, ultimately leading to cell death.

IGF-1 protects cardiomyocytes by stabilizing mitochondria and reducing ROS damage. Pi et al^[45]^ found that IGF-1 protects rat cardiomyocytes subjected to H/R stress by preventing impairment of mitochondrial bioenergetic functions and membrane potentials. Their research results indicated that IGF-1 stabilizes mitochondrial function, making cardiomyocytes more resistant to H/R injury. Various research has indicated that IGF-1 reduces angiotensin II^[46]^ or hyperglycemia-induced ROS generation.^[47]^ In contrast, IGF-1 signaling regulates cardiac PTP. IGF-1 has been described to restrain Ca^2+^-sensitive mitochondrial swelling^[48]^ and decrease Ca^2+^-stimulated mitochondrial cytochrome c secretion, indicating that IGF-1 signaling regulates cardiac PTP. Glycogen synthase kinase-3, a potential downstream target of IGF modeling channels, has been reported to modulate PTP opening in cardiomyocytes.^[49]^ These beneficial effects of IGF-1 would make mitochondria more protective of the myocardium against oxidative stress. Davani et al^[50]^ found that in an experimental model of myocardial ischemic injury, IGF-1 treatment improved myocardial tissue structure and function while also maintaining the mitochondrial DNA to nuclear DNA ratio in the mouse heart following I/R. Lai et al^[51]^ established an animal model demonstrating that IGF-1 prevented a decrease in mitochondrial electrochemical gradient loss and membrane depolarization. In addition, IGF-1 prevented mitochondrial DNA injury after I/R injury and promoted mitochondrial ATP synthesis in rat cardiomyocytes. These results indicate that IGF-1 provides benefits to cardiac mitochondria. In summary, the involvement of mitochondria in IGF-1-induced defense of cardiomyocytes against I/R injury may provide novel insights into the clinical diagnosis and treatment of MIRI.

### 4.3. IGF-1 signaling pathway

The IGF-1 signaling pathway is strongly implicated in the regulation of cell growth and survival.IGF-1 initiates signaling cascades by activating IGF-1R. Specific tyrosine residues of IGF-1R undergo autophosphorylation upon ligand-receptor binding, thereby activating 2 downstream signaling pathways: the PI3K/Akt and Ras/Raf/ERK cascades.^[52]^ The PI3K/Akt pathway begins with tyrosine autophosphorylation of the IGF-1Rb subunit and is implicated in cell growth, metabolism, and antiapoptotic responses.^[53]^ The Ras/Raf/ERK pathway plays an important role in regulating cell survival, growth, and differentiation. IGF-1 synergistically acts with the Akt/mTOR (mammalian target of rapamycin) pathway through the Ras/Raf/ERK pathway to induce cell proliferation and growth.^[54]^ Additionally, IGF-1 directly or indirectly regulates many signaling pathways in MIRI. For instance, IGF-1 can affect mitochondrial function and reduce mitochondrial damage, thereby reducing the severity of MIRI. Studies have demonstrated that treating rats with systemic IGF-1 upregulates Bcl-xL expression, downregulates pro-apoptotic Bax proteins in mitochondria, and significantly reduces cytochrome c release and PTP opening in I/R injury.^[48]^ Furthermore, high expression of Bcl-Xl influences mitochondrial membrane potential, swelling the matrix and preventing cell death.^[55]^ IGF-1 also plays a significant role in oxidative stress response. As an active player in oxidative stress response, IGF-1 regulates mitochondrial function dependent on the PI3K/Akt pathway and reduces mitochondrial ROS generation in Huntington disease knock-in striatal cells.^[56]^ The IGF-1 pathway and apoptosis are closely related, as the antiapoptotic effects of IGF-1 resists the effects of myocardial I/R damage by acting on the PI3K/Akt pathway.^[29]^ IGF-1 inhibits cellular autophagy, ameliorating stimulus-induced cardiomyocyte autophagy by raising ATP expression and promoting mitochondrial metabolism, including mitochondrial Ca^2+^ ingestion and oxygen consumption, via the AMP-activated protein kinase (AMPK)/mTOR and Akt/mTOR axes.^[57]^ In addition, IGF-1 activates multiple signaling channels, such as GSK-3/mitochondrial PTP, Ras/Raf/ERK, PI3K/AKT, Bcl-2, and mTOR, which are involved in modulating a variety of cellular functions, thereby alleviating the severity of MIRI.

### 4.4. IGF-1 and cardiomyocyte apoptosis

Apoptosis, also known as type I programmed cell death, is involved in physiological processes such as cell turnover, embryonic development, and the removal of inflammatory cells. It is well-established that various stimuli, such as hypoxia, ischemia, and reperfusion, as well as oxidative stress, are involved in and induce apoptosis in cardiomyocytes.^[58]^ Previous studies have indicated that apoptotic cell death is the primary form of cardiomyocyte death during MIRI.^[59,60]^ Reperfusion seems to speed up the time to apoptosis compared to permanent occlusion. The antiapoptotic action of IGF-1 is facilitated by IGF-1R and the accompanying activation of the Ras/Raf/MEK/ERK and PI3K/Akt/mTOR signaling channels. Akt functions by activating antiapoptotic targets or inactivating pro-apoptotic agents to achieve its antiapoptotic properties. Well-known targets of Akt include the glycogen synthase kinase (GSK)-3b, cAMP-responsive element-binding protein, nuclear factor-kB, and bcl-2 family member Bcl-2-associated death promoter (BAD).^[61]^ The MAPK protein family is essential for IGF-1-dependent signaling. In particular, signal-regulated extracellular kinases ERK1 and ERK2 are involved in protection from apoptosis.^[62]^ BAD performs its apoptotic function through heterodimerization of Bcl-2 and Bcl-XL bound to mitochondria, which neutralizes its protective function and promotes cell death. After IGF-1 stimulation, Akt phosphorylates BAD depending on PI3K, and ERK1 and ERK2 phosphorylate depending on MEK1.^[61]^ The antiapoptotic role of IGF-1 relies on the PI3K and MEK1 pathways, which further activate the transcription factor cAMP-responsive element-binding protein and induce increased expression of the antiapoptotic factor bcl-2 in cardiomyocytes.^[62]^ These processes are associated with mitochondrial energetics and the mitochondrial apoptotic pathway.^[48]^ This may partly explain how IGF-1 protects the myocardium during the later stages of reperfusion injury. The inducible activity of Akt results in the expansion of the antiapoptotic transcription factor NF-kB, which reduces the activities of the pro-apoptotic molecule p53. Decreased expression of p53 is important for preventing apoptosis, as p53 leads to decreased expression of IGF-1R and up-regulation of pro-apoptotic Bax proteins. GSK-3b, a protein associated with triggering apoptosis in terminally differentiated neurons, represents another protective pathway for Akt activation through its inhibition.^[63]^ IGF-1 has been shown to have protective and antiapoptotic properties in various models of myocardial ischemia and infarction, as well as in isolated cardiomyocytes subjected to ischemic or oxidative stress.^[64]^ In addition, the overexpression of IGF-1 may protect cardiomyocytes from apoptosis after ischemia or postischemic reperfusion. In recent years, researchers have discovered that IGF-1 has insulin-like effects and mediates growth hormone production, and inhibits various types of apoptosis.^[65]^ IGF-1 suppresses apoptosis by associating with surface receptors on particular target cells. Although the mechanism by which IGF-1 regulates apoptosis inhibition has not been completely clarified, numerous unidentified signaling channels and regulatory miRNAs may still play a role in it. IGF-1 can be considered as a potential target for the treatment of MIRI.

### 4.5. IGF-1 and autophagy

Autophagy exists at a low foundational level in most cells to maintain homeostatic functions, including protein and organelle turnover.^[66]^ Autophagy can be triggered under various stress conditions such as hypoxia, oxidative stress, nutrient starvation, and growth factor deficiency.^[67]^ The induction of autophagy is mainly controlled at 2 key nodes: mTOR and AMPK.^[68]^ Robust autophagy is associated with a wide range of stressors that induce cardiac lesions, including increased afterload, hypoperfusion, and I/R injury.^[69]^ Autophagy has different roles depending on the degree of induction and the conditions under which it occurs, and it can either promote or antagonize disease pathogenesis. When cells are starved during ischemia, the induction of autophagy is protective. However, autophagy is maladaptive under severe afterload stress.^[70]^ IGF-1 has also been observed to have an inhibitory effect on autophagy. In particular, long-term IGF-1 exposure reduces cell viability and autophagy.^[71]^ Gu et al^[72]^ found that serum abstinence induced autophagy in mitochondria with deleterious mutations in mitochondrial DNA, whereas the addition of IGF-1 prevented the effect of serum abstinence on mitochondrial autophagy. Autophagy in bovine mammary epithelial cells is complex. IGF-1 is involved in the suppression of autophagy, and the mTOR kinase is a pivotal mediator of the process.^[73]^ Moreover, IGF-1 inhibits autophagy in plaque-derived vascular smooth muscle cells through Akt-dependent suppression of LC3 expression.^[74]^ Additionally, IGF-1 deficiency increases cardiac AMPK activity during starvation, suggesting that the energy-conserving efficacy of IGF-1 modulates starvation-induced cardiac autophagy.^[57]^ Taneike observed that a short course of IGF-1 treatment protected the cardiac against the stress of nutritional deprivation.^[75]^ In contrast, IGF-1 promotes autophagy in Purkinje neurons and H9c2 cardiomyocytes. IGF-1/PI3K increases the accumulation of autophagic vacuoles and promotes autophagic cell death in cardiomyocytes during glucose deprivation.^[76]^ Bains et al^[77]^ proposed that IGF-1 plays a novel role in protecting Purkinje neurons from autophagy-related cell death by enhancing the efficiency of autophagy downstream of induction. Autophagy also ameliorates I/R damage via miRNAs. Su et al^[78]^ found that inhibition of the long noncoding RNAs (lncRNA) TUG1 upregulated miR-142-3p, thereby alleviating IR injury by targeting HMGB1 and Rac1-induced autophagy. In summary, IGF-1 may promote or inhibit autophagy; however, further research is needed to clarify the regulatory effect of IGF-1 in MIRI.

### 4.6. Crosstalk between IGF-1 and noncoding RNAs

miRNAs belongs to a class of evolutionarily conserved, endogenous, noncoding small molecule RNAs with approximately 21 to 24 nucleotide. It has been found that miRNAs mainly bind to target mRNAs through their 3′ untranslated regions, thereby inhibiting or promoting DNA translation and regulating gene expression at the transcriptional level. Currently, convincing findings indicate that miRNAs are able to regulate a wide range of biological functions, including cell survival, differentiation, autophagy, and apoptosis.^[79,80]^ miRNAs play a pivotal role in MIRI. Wang et al^[81]^ showed that MiR-29a and Let7 could improve MIRI by influencing apoptosis through the regulation of IGF-1 in animal experiments. Fan et al^[82]^ showed that targeting IGF-1 to downregulate miR486-5p significantly increased hypoxia-induced cardiomyocyte survival and inhibited hypoxia-induced myocyte apoptosis. Zhan et al^[83]^ concluded that “the activation of the PI3K/Akt signaling channel further suppressed hypoxia-induced oxidative stress levels and apoptosis in AC16 cells via SnHG1/miR-450b-5p/IGF-1 axis dependence.” Down-regulation of miR-320 suppresses myocardial apoptosis and protected he myocardium from MIRI by targeting IGF-1.^[84]^ miRNAs related to IGF-1 also play important regulatory roles in other tissues. Fu et al^[85]^ revealed that miR-199a-3p is implicated in estrogen-mediated autophagy via the IGF-1/mTOR pathway in osteocyte-like MLO-Y4 cells. In addition, miR-28-5p inhibited hepatic CSC expansion through direct regulation of IGF-1.^[86]^ Zhu et al^[87]^ state that miR-129 regulates axonal regeneration by modulating IGF-1 during peripheral nerve injury. Das et al^[88]^ found that miR-214 decreased IGF-1 repression and the signaling of downstream mTORC1 in renal cancer cells. Despite the increasing number of known miRNAs related to IGF-1, their role in cardiovascular disease, particularly MIRI, is poorly understood.

LncRNAs longer than 200 nucleotides in length and lacking the ability to encode proteins play essential roles in various biological processes, including cell survival, differentiation and apoptosis.^[89]^ They exert both positive or negative influence on the expression levels of protein-coding genes through a variety of modes and are strongly associated with cardiovascular disease.^[90]^ Recent evidence underscores the significant involvement of lncRNAs in the regulation of myocardial I/R regulation. For instance, Yu et al^[91]^ investigated the effect of lncRNA AK139328 on autophagy and apoptosis in MIRI. AK139328 knockdown alleviated myocardial I/R injury and suppressed cardiomyocyte apoptosis and autophagy in diabetic mice. LncRNA AK139328 directly modulated miR-204-3p. Down-regulation of lncRNA AK139328 dramatically enhanced miR-204-3p expression and suppressed autophagy in cardiomyocytes, resulting in attenuation of MIRI. Tong et al^[92]^ evaluated the effect of lncRNA LSINCT5 in MIRI regulation and concluded that lncRNA LSINCT5 was upregulated during acute myocardial infarction development. Moreover, they suggested that LSINCT5 may regulate MIRI through the PI3K/AKT modeling channel and the LSINCT5/miR-222 axis. However, the relationship between IGF-1 and lncRNAs remains incompletely elaborated. Zhang et al^[93]^ results indicate that lncRNA NR2F1-AS1 enhances IGF-1 in breast cancer cells via sponge miRNA-338-3p and activates IGF-1R and ERK pathways in human umbilical cord vascular endothelial cells, further promoting breast cancer angiogenesis. While ample evidence highlights the association between IGF-1 and lncRNAs in different disorders,^[94]^ their specific mechanisms of action in MIRI require further investigation. In-depth research could lead to new ideas for the future diagnosis and treatment of MIRI.

## 5. IGF-1-controlled factors confer myocardial protection against MIRI

Some research suggests that certain substances can alleviate MIRI, and IGF-1 mediates its therapeutic effects. Resveratrol is a natural phenol with a variety of pharmacological effects. He et al^[95]^ demonstrated that resveratrol administration inhibited the acceleration of the NF-κB signaling process and reduced the concentration of IGF-1, NGF, and IL-6 in the myocardium with acute myocardial infarction, which resulted in a protective effect against MIRI. Thyroid hormone is inextricably linked to the regulation of cardiac mechanical function and electrophysiological properties. Zeng et al^[96]^ demonstrated that T3 preconditioning protects cardiomyocytes from H/R-induced damage through IGF-1, which in turn activates the PI3K/Akt signaling pathway. Suppressor of cytokine signaling 2 is a member of the family of cytokine signaling suppressor proteins, which exerts negative regulatory effects on various cytokine-mediated biological processes. Sheng et al^[97]^ suggested that overexpression of suppressor of cytokine signaling 2 may downregulate IGF-1 expression, thereby exacerbating MIRI in patients with type 2 diabetes. FoxO3a, a member of the FoxO subfamily of forkhead transcription factors, can be phosphorylated by Akt. Qi et al^[98]^ indicate that IGF-1 blocks caspase-3 activation in ischemia-reperfusion-induced cardiac microvascular endothelial cells and inhibits FoxO3 activation through activation of Akt. The FoxO3a pathway is partially implicated in I/R damage in CMECs by regulating apoptosis and cell cycle arrest. Brain-derived neurotrophic factor (BDNF) is involved in mitochondrial bioenergetics, exercise-induced neurogenesis, and regulation of energy homeostasis in the brain.^[99]^ BDNF and its related myosin-related kinase receptor B (TrkB) can influence the nerves and blood vessels of the heart. Myocyte-borne BDNF is important for myocardial contractility,^[100]^ metabolism,^[101,102]^ and the response to ischemia.^[103]^ IGF-1 enhances TrkB expression and increases the ability of BDNF to induce ERK1/2 phosphorylation.^[104]^ Additionally, myocyte-specific BDNF deletion exacerbates I/R injury.^[103]^ However, further investigation is required to determine if BDNF directly inhibits MIRI via IGF-1.

## 6. IGF-1 and its role in ischemic preconditioning (IPC) and ischemic post-conditioning (I-postC)

Ischemic preconditioning (IPC) involves transient nonfatal ischemia and reperfusion before fatal ischemia occurs. IPC has been reported to confer protection against subsequent I/R injury.^[105]^ Lu et al^[106]^ demonstrated that the inherent survival mechanism of stem cells against ischemic injury in IPC is enhanced by the coordinated activation of the protein kinase Ca-Erk1/2. This activation improves stem cell survival in oxygen-deprived, glucose-deprived, and posttransplant experimental infarcted hearts. The protective mechanism underlying IPC is mediated by IGF-1. Zhang et al^[107]^ demonstrated that IPC activates IGF-1 and enhances its mRNA expression, ultimately inhibiting ischemia- and hypoxia-induced apoptosis. It is well known that ROS production and consequent oxidative stress increase apoptosis, necrosis, and inflammation and impair mitochondrial function. IPC has also been shown to attenuate I/R-induced defects and protect against oxidative stress. The mitochondrial K^+^-ATP channel acts as an effector and a trigger of IPC. ROS released by mitochondria during IPC leads to the opening of mitochondrial K+-ATP pathway, which is protective.^[108]^ Stabilization of mitochondria and reduction of ROS damage is dependent on IGF-1 and is protective for cardiomyocytes against MIRI.^[45]^ Earlier studies have demonstrated that PKC activation is a critical intracellular signal for IPC induction,^[109]^ with IGF-1 activating PKC-dependent protein synthesis in adult rat cardiomyocytes. These findings suggest that PKC can target IGF-1 to produce cardioprotective effects.^[110]^ Overall, IPC represents the most powerful intrinsic protective mechanism against I/R injury. These studies suggest that IGF-1 is activated during IPC and is protective against subsequent I/R injury.

I-postC has also been reported to improve myocardial function to relieve MIRI. The concept of I-postC, first proposed in 2003,^[111]^ involves one or more short-duration repeated myocardial ischemia and reperfusion cycles preceding a short-duration MIRI. I-postC induces an intrinsic protective effect against myocardial injury in response to prolonged ischemia, thereby reducing infarct size and protecting cardiac function.^[112]^ I-postC has been found to be as equally effective as IPC in providing protection against MIRI in the heart. Investigating into the model signaling molecules involved in I-PostC and IPC IPC identified a common cardioprotective pipeline involving protein kinases such as GSK3b, Akt, and Erk1/2. The “reperfusion injury salvage kinase” (RISK) pathway was recruited during MIRI,^[113,114]^ along with antiapoptotic machinery pathways, including the phosphorylation of BAD.^[115]^ Fujio et al^[16]^ suggested that the PI3K-dependent stimulation of Akt is a critical link in the IGF-1 signaling channel that prevents cardiomyocyte death. Akt promotes cardiomyocyte survival and protects the heart from I/R injury in mice. Liao et al^[29]^ showed that IGF-1 protects cardiomyocytes from I/R damage by suppressing cardiomyocyte apoptosis in vivo. In summary, IGF-1 plays critical roles in IPC and I-postC, making it an attractive candidate for MIRI treatment.

## 7. Future perspectives

**Figure 1.**
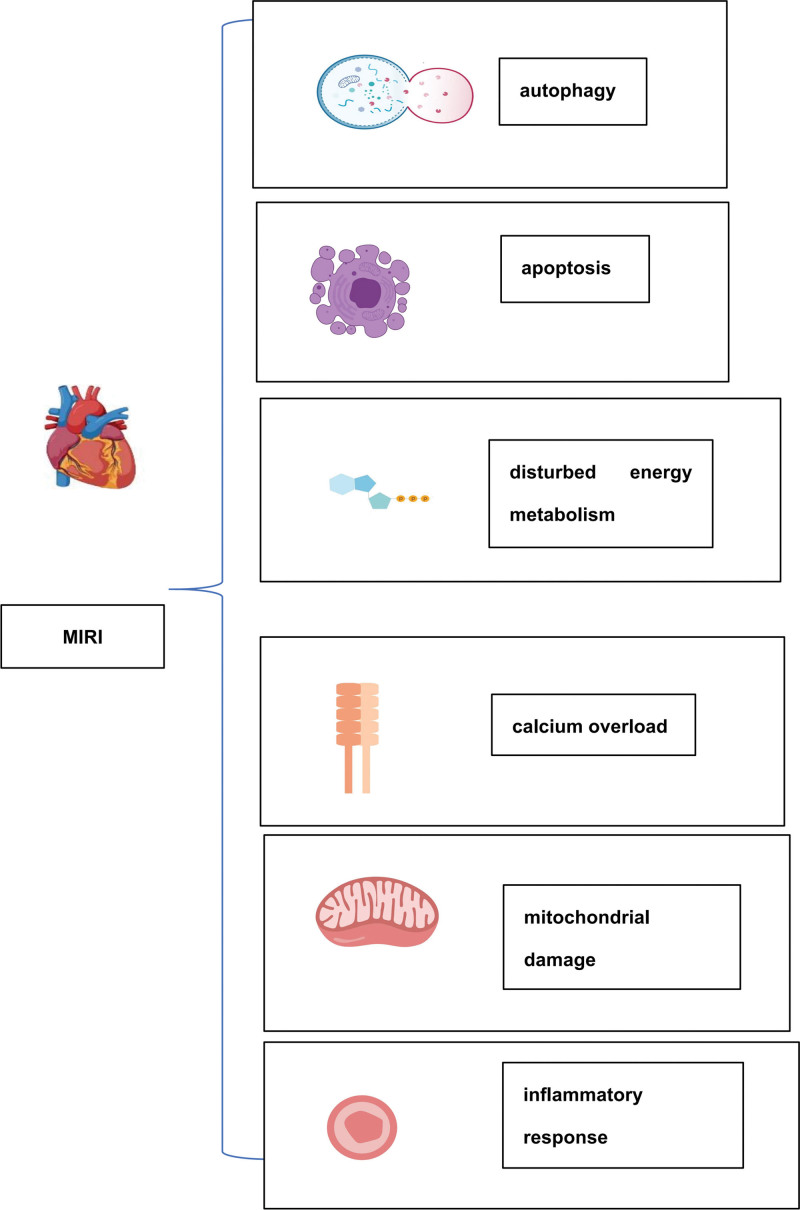
Pathophysiologic mechanisms involved in MIRI. Calcium overload, oxidative stress, mitochondrial damage, disturbed energy metabolism, inflammation, apoptosis, and autophagy are collectively involved in the pathophysiological process of MIRI. MIRI = myocardial ischemia-reperfusion injury.

**Figure 2.**
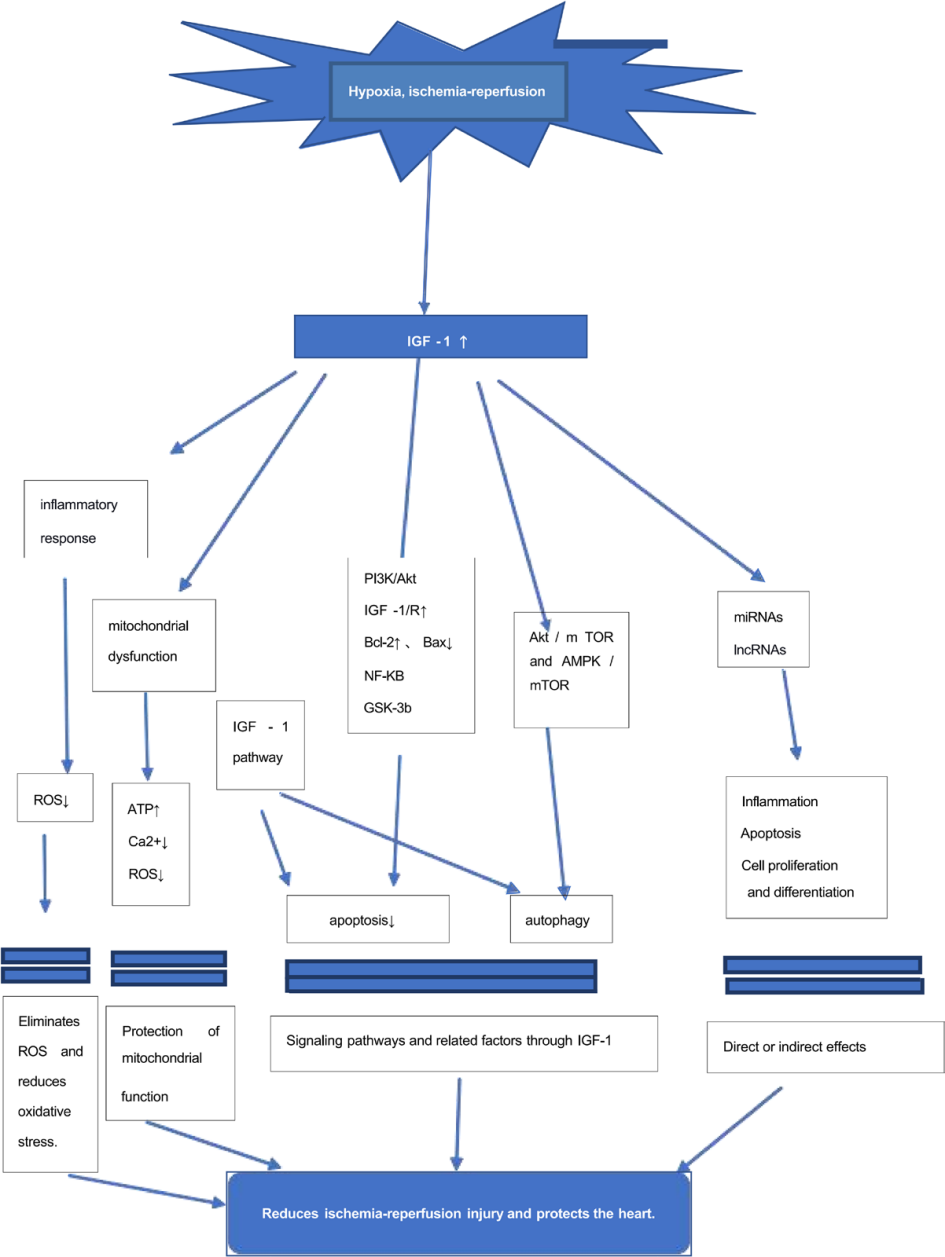
Role of IGF-1 in MIRI. Upward arrows indicate enhancement, while downward arrows indicate inhibition. BAD = bcl-2-family member; IGF-1 = insulin-like growth factor 1; (GSK)-3b = glycogen synthase kinase; lncRNAs = long noncoding RNAs; MIRI = myocardial ischemia-reperfusion injury; miRNA = microRNA; NF-kB = the transcription factors nuclear factor-kB; ROS = reactive oxygen species.

**Figure 3.**
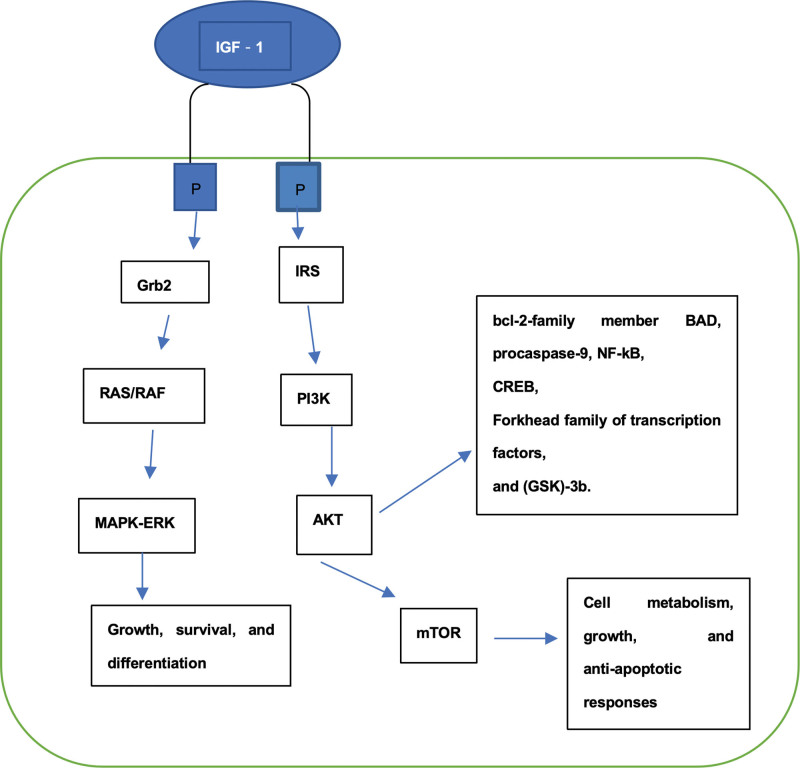
Signaling pathways associated with IGF-1. IGF-1= insulin-like growth factor 1.

Cardiovascular and cerebrovascular diseases endanger human health and lives. The main treatment for ischemic diseases involves the recovery of blood perfusion, relief of tissue hypoxia, and addressing nutrient shortage. The mechanisms of action underlying MIRI have been extensively studied and involved multiple contributing agents. Cardiomyocyte death throughout the pathophysiologic process may result from multiple synergistic mechanisms (Fig. 1). A wide range of molecular targets with cardioprotective roles have been identified in MIRI,^[116,117]^ with IGF-1 being one of the most promising targets.^[16]^ IGF-1 mitigates MIRI through various complex mechanisms (Fig. 2), including cell survival, mitochondrial function, apoptosis, and cellular autophagy. Additionally, IGF-1 interacts with different noncoding RNAs and signaling pathways (Fig. 3), although these interactions have been fully characterized. IGF-1 can regulate several target genes and activate different pathways that play an integral role in various stages of MIRI progression. Notably, other mechanisms involved in regulating the progression of MIRI pathophysiology and disease regression need to be explored in the future, as the latest data primarily stems from animal experiments and lacks sufficient clinical data. Therefore, results from a wide range of randomized controlled trials and clinical studies are needed to support this hypothesis. Consequently, further investigation into the effects of IGF-1 in the pathophysiology of MIRI is necessary for future MIRI prevention and treatment. Developing therapies targeting the molecular mechanisms underlying the development of MIRI is critical to for improving clinical outcomes for patients.

## Acknowledgments

We would like to thank Editage (www.editage.com) for English language editing.

## Author contributions

**Conceptualization:** Zhenrong Yan, Ziyang Xing.

**Project administration:** Qiyu Sun.

**Supervision:** Qiyu Sun.

**Writing – original draft:** Zhenrong Yan.

**Writing – review & editing:** Zhenrong Yan, Ziyang Xing, Tingyun Xue, Jiaye Zhao, Guangmei Li, Liwenjing Xu, Qiyu Sun.
